# Supplementary material for: Prevalence of nasopharyngeal Streptococcus Pneumoniae carriage in infants: A systematic review and meta-analysis of cohort studies and randomized controlled trials
Source: PLoS One. 2024 Dec 18;19(12):e0315461. doi: 10.1371/journal.pone.0315461 (PMC11654947; doi:10.1371/journal.pone.0315461)
Supplement: S1 Table — (DOCX) [file pone.0315461.s002.docx]

**S1 Table. Search strategy**

| **Pubmed** | **Web of Science** | **Science Direct** | **Scopus** |
| --- | --- | --- | --- |
| **Criteria: Search in All Fields.**  **Filter: Language: English, Species: Human**  **Search date: March – June 2024**  (("Streptococcus pneumoniae" OR "pneumococcal") AND "carriage" AND ("children under 2 years" OR "infants"))- 273 | **Criteria: Search in All Fields.**  **Filter: include only “Articles”**  **Search date: March – June 2024**  ( ( "Streptococcus pneumoniae" OR "pneumococcal" ) AND "nasopharyngeal carriage" AND ( "children under 2 years" OR "infants" ) ) - 235 | **Criteria: Search in All Fields.**  **Filter: Title abstract keyword search**  **Search date: March – June 2024**  ( ( "Streptococcus pneumoniae" OR "pneumococcal" ) AND "nasopharyngeal carriage" AND ( "children under 2 years" OR "infants" ) ) - 61 | **Criteria: Search in All Fields.**  **Filter: Limit to Document type: Article. Language: English.**  **Search date: March – June 2024**  ( ( "Streptococcus pneumoniae" OR "pneumococcal" ) AND "nasopharyngeal carriage" AND ( "children under 2 years" OR "infants" ) ) - 514 |
