# Supplementary material for: Prevalence of nasopharyngeal Streptococcus Pneumoniae carriage in infants: A systematic review and meta-analysis of cohort studies and randomized controlled trials
Source: PLoS One. 2024 Dec 18;19(12):e0315461. doi: 10.1371/journal.pone.0315461 (PMC11654947; doi:10.1371/journal.pone.0315461)
Supplement: Attachment — Submitted filename: Response to the reviewers.docx [file pone.0315461.s005.docx]

**Response to the reviewers**

**Journal requirements:**

1. Please ensure that your manuscript meets PLOS ONE's style requirements, including those for file naming. The PLOS ONE style templates can be found at

https://journals.plos.org/plosone/s/file?id=wjVg/PLOSOne_formatting_sample_main_body.pdf and

<https://journals.plos.org/plosone/s/file?id=ba62/PLOSOne_formatting_sample_title_authors_affiliations.pdf>

**Authors reply:** The manuscript and the title page have been revised to follow the journal guidelines. The author information was deleted from the first page, and the title of the manuscript was revised.

2. In the online submission form, you indicated that [Data used for the analysis in this study is available from the corresponding author upon reasonable request].

All PLOS journals now require all data underlying the findings described in their manuscript to be freely available to other researchers, either 1. In a public repository, 2. Within the manuscript itself, or 3. Uploaded as supplementary information.

This policy applies to all data except where public deposition would breach compliance with the protocol approved by your research ethics board. If your data cannot be made publicly available for ethical or legal reasons (e.g., public availability would compromise patient privacy), please explain your reasons on resubmission and your exemption request will be escalated for approval.

**Authors reply:** All requested data is provided as Supplementary Tables (S2 Table and S3 Table).

3. As required by our policy on Data Availability, please ensure your manuscript or supplementary information includes the following:

A numbered table of all studies identified in the literature search, including those that were excluded from the analyses.

For every excluded study, the table should list the reason(s) for exclusion.

If any of the included studies are unpublished, include a link (URL) to the primary source or detailed information about how the content can be accessed.

A table of all data extracted from the primary research sources for the systematic review and/or meta-analysis. The table must include the following information for each study:

Name of data extractors and date of data extraction

Confirmation that the study was eligible to be included in the review.

All data extracted from each study for the reported systematic review and/or meta-analysis that would be needed to replicate your analyses.

If data or supporting information were obtained from another source (e.g. correspondence with the author of the original research article), please provide the source of data and dates on which the data/information were obtained by your research group.

If applicable for your analysis, a table showing the completed risk of bias and quality/certainty assessments for each study or outcome.  Please ensure this is provided for each domain or parameter assessed. For example, if you used the Cochrane risk-of-bias tool for randomized trials, provide answers to each of the signalling questions for each study. If you used GRADE to assess certainty of evidence, provide judgements about each of the quality of evidence factor. This should be provided for each outcome.

An explanation of how missing data were handled.

This information can be included in the main text, supplementary information, or relevant data repository. Please note that providing these underlying data is a requirement for publication in this journal, and if these data are not provided your manuscript might be rejected.

**Authors reply:** all requested data that were missing from the manuscript are provided as Supplementary Tables (S2 Table and S3 Table).

We have also revised the data availability statement, and added the S2 Table and S3 Table Titles in lines 520 – 521: **S2 Table. Detailed study selection process results**

**S3 Table. Comprehensive data extraction summary**

Lines 542 – 544: **Availability of data**

All data necessary to replicate the study’s findings is provided in supplemental materials.

**Reviewer #1:**

1. This systematic review approaches the pneumococcal carriage state for a much younger age group with very interesting results. The manuscript will benefit from significant shortening especially the results section for figures. My main criticism would be the language highly equating the carriage with clinical illness. Especially in infants less than 6 months of age the clinical pneumococcal disease is not significant.

**Authors reply:**

The authors sincerely appreciate the reviewer’s insightful feedback regarding our manuscript. We recognize the importance of clarity in differentiating between pneumococcal carriage and clinical illness, particularly for infants under six months, where the incidence of clinical disease is indeed low.

We have taken steps to address your concerns by substantially shortening the results section and restructuring the presentation to improve readability and coherence. The number of figures presented is integral to our objective of exploring the prevalence of non-invasive pneumococcal carriage (NSPC) among infants in their first two years, while also considering variations by vaccine type and country income levels.

In our revisions, we have highlighted significant disparities in NSPC prevalence across different economic contexts, emphasizing the need for a nuanced understanding of these factors, and have discussed this issue in more detail in the revised discussion section. All modifications have been clearly marked in the results and discussion sections of the manuscript, particularly between lines 219 - 389 & 442 -508. We hope that these changes will satisfactorily address the reviewer’s comments and enhance the overall clarity and impact of our work.

2. Under methods, please specify the analysis period. Please also provide references for the statistical methods used. Please also explain how the age categories were calculated based on the review as some publications may not have reported this.

**Authors reply**: The authors appreciate the reviewer’s valuable feedback, and offer the following changes:

1. We have added the following statement in lines 103 – 104 to specify the analysis period: No restrictions were imposed on the publication dates of studies
2. The following reference was added to the statistical methods: Subgroup analysis based on vaccination status and country income level was used to calculate the pooled mean prevalence of NSPC for each specified age group, along with 95% confidence intervals (95% CI), using a random-effects model for meta-analysis in RStudio software with the “meta” and “metafor” packages [18].
18. Harrer M, Cuijpers P, Furukawa TA, Ebert DD. Doing Meta-Analysis with R: A Hands-On Guide. Boca Raton, FL and London: Chapman & Hall/CRC Press.; 2021. Available: <https://bookdown.org/MathiasHarrer/Doing_Meta_Analysis_in_R/pooling-es.html>

3.The authors appreciate the reviewer’s valuable feedback regarding the categorization of age groups. We have derived the age categories presented in this review based on a consensus among the three authors responsible for data extraction, after a careful review of existing literature. In instances where studies reported NSPC rates in age brackets that differed significantly from those used in our meta-analysis (e.g., 3–12 months or 12–36 months), we excluded these studies to maintain consistency across age groupings. A summary of all excluded articles with non-aligned age ranges is provided in Supplemental Table S2 for further clarity. We hope this approach clarifies the methodology used for age categorization and meets the reviewer’s expectations.

**Reviewer #2:** In this systematic review and meta-analysis, the authors determined the prevalence of nasopharyngeal carriage of Streptococcus pneumoniae (NSPC) among infants during their first two years of life, stratified by month of age. They also did subgroup analysis for the carriage rates among infants who received different vaccines and also based on country income levels. The authors reported that the pooled mean prevalence of NSPC was highest in the 7 to 9-month age group. Additionally, the prevalence was higher among infants aged 4 to 6 months and 7 to 9 months, across all vaccine groups. Furthermore, the study found that low-income countries exhibited the highest NSPC rates across all age groups.
1. Although the manuscript has considered all the necessary parameters required for performing meta-analysis like PRISMA guidelines, risk of bias assessment, publication bias assessment, however, it needs to be better structured so that one can follow it easily.

**Authors reply**: the authors acknowledge the reviewer’s insightful feedback. The results and discussion section have been extensively revised, and in the results section, we have added the explanatory paragraph on the structure of the results presentation in lines 219 – 223: In this section, we present the prevalence of nasopharyngeal SP carriage in infants during the first two years of life, stratified by vaccine type and country income level. The data are organized by age intervals as follows: 0 months, 1–3 months, 4–6 months, 7–9 months, 10–12 months, 13–18 months, and 19–24 months.

2. In the title, it is Streptococcus pneumoniae carriage, “e” is missing from pneumoniae. Moreover, it should be in italics.

**Authors reply:** the authors acknowledge the reviewer’s comment and agree with the reviewer. The revised manuscript title is as follows: Prevalence of nasopharyngeal Streptococcus *pneumoniae* carriage in infants: a systematic review and meta-analysis of cohort studies and randomized controlled trials

3. In material and methods section, authors should mention PICOS in the search strategy. Authors have mentioned risk of bias, instead they can add study quality assessment. Moreover, authors have not mentioned anything about the restrictions imposed, if any.

**Authors reply:** the authors acknowledge the reviewer’s thoughtful comments and agree with the reviewer.

1. The authors have revised the search strategy description, and have added the following description in lines 94 – 100: The search was structured according to the Population, Intervention, Comparator, Outcomes, and Study Design (PICOS) framework as follows: Population (P): infants and children under two years of age; Intervention (I): not applicable; Comparator (C): not applicable; Outcomes (O): nasopharyngeal carriage; and Study Design (S): cohort studies and randomized clinical trials (RCTs).
2. The risk of bias assesment and the assessment of the quality of the included studies were performed by the same tool. The authors changed the subheading to reflect this important omission in line 146: **Risk of bias and study quality assessment**
3. Restrictions imposed to the search strategy are provided in the Full search strategy as Supplemental Table.

4. In result section, in line no. 207, India and Bangladesh should be removed as the no. are not getting matched, India has got repeated and Bangladesh is not in the table.

**Authors reply:** The authors acknowledge the reviewer’s thoughtful comment and agree with the reviewer. The study by Apte, 2021 provided data for children in India and Bangladesh, and the Table 1 was missing this information. We have revised the Table 1 and added the Bangladesh to the list.
5. In the result section, Data on the NSPC at birth comprises of vaccine groups which includes PCV 7 and PCV 10 group in line no. 238, while in the diagram it is PCV 7 and PCV 13. Please clarify.

**Authors reply:** The authors acknowledge the reviewers thoughtful comment, and agree with the reviewer. This was an omitted typo from the authors side, the groups description was changed to PCV7 and PCV13, as reflected in the Figure 2. The complete data extraction table is added to the manuscript as supplemental S3 Table, and the data is also provided there to support the clarification.
6. In the subgroup analysis of the vaccine group, authors should talk about dosage of the vaccination as well as serotypes that are covered. Infact, in my opinion authors have categorized groups taking dosage into account. If that is the case, it should be more elaborative and clear. If not than they should specify how they have categorized the groups. Authors should add that in supplementary information.

**Authors reply:** The authors acknowledge the reviewer’s thoughtful comments and agree with the reviewer. The dosage of the vaccination was also extracted as presented in S3 Table (data extraction table), but the data was too heterogeneous and incomplete to analyze the vaccination based on the dosage. Thus, we did not include any information on the analysis based on the vaccination dosage to the methods section.

We have also expanded the limitation section of the manuscript to reflect that we did not investigate the serotypes in Lines 503 – 508: Lastly, this review did not analyze serotype distribution within Streptococcus *pneumoniae*, which is crucial for future vaccine strategy development. Understanding the prevalence of specific serotypes is essential to tailoring pneumococcal vaccine formulations effectively, especially for low-income regions where serotype diversity may significantly influence vaccine effectiveness.
7. Discussion section is poorly written, it needs to be more structured and organized. Authors should discuss about the vaccine type which is more effective, and serotypes covered by the vaccines. Also, whether the observed prevalence of NSPC is due to vaccine or non-vaccine type serotypes.

**Authors reply:** The authors acknowledge the reviewer’s thoughtful comments and agree with the reviewer. We have revised the discussion to follow the following outline: main findings of the study, vaccine effectiveness and serotype dynamics, disparities in NSPC prevalence, policy implications, limitations and conclusion as presented in the updated discussion section. The changes are highlighted starting from line 440 – 508:

This systematic review and meta-analysis provide a comprehensive assessment of the prevalence of NSPC in healthy infants during their first two years of life. Our findings reveal a notable prevalence of NSPC across diverse vaccine groups and income settings, indicating a sustained rate of pneumococcal carriage in this vulnerable age group. The pooled prevalence from cohort studies and randomized controlled trials suggests that NSPC remains prevalent in infants aged 4 to 6 months and 7 to 9 months, irrespective of vaccination status, with a general decline observed afterward.

Vaccination has profoundly impacted pneumococcal serotype carriage, with the introduction of PCVs leading to a significant reduction in vaccine serotypes among infants [69]. However, non-vaccine serotypes continue to persist, albeit generally with lower virulence, and still contribute to NSPC, underscoring the need for ongoing serotype surveillance due to serotype replacement. The overall reduction in vaccine-type carriage represents a public health milestone in lowering invasive IPD risks, such as pneumonia, meningitis, and sepsis, in vaccinated populations [70].

The evolution of PCV formulations has aimed to address serotype replacement. Following the WHO’s recommendation in 2010 to transition from PCV7 to PCV13, which includes additional serotypes (1, 3, 5, 6A, 7F, and 19A), significant strides were made in reducing IPD. However, as non-vaccine serotypes emerge and become more virulent, the development and potential widespread implementation of higher-valent vaccines, such as the 20-valent PCV (PCV20), is actively under consideration. Studies from high- and upper-middle-income countries indicate that PCV20, covering additional serotypes (8, 10A, 11A, 12F, 15B, 22F, and 33F), is not only effective but also cost-efficient, especially in pediatric populations using the 2+1 dosing schedule [71–77]. Recent systematic reviews suggest that transitioning from PCV13 to PCV20, rather than to the 15-valent PCV (PCV15), would result in greater cost savings by effectively addressing serotype replacement and reducing the economic burden associated with pneumococcal diseases [78].

Our analysis highlights significant disparities in NSPC based on country income level. Infants in low-income countries exhibit higher NSPC rates, with 74.22% (95% CI [54.25; 87.49]) prevalence among 19- to 24-month-olds. Factors such as limited vaccination coverage, crowded living conditions, and socio-economic constraints contribute to this elevated carriage rate. Studies, including work by Adegbola and colleagues, show a pre-vaccine NSPC prevalence as high as 93.4% in children up to 48 months in low-income settings [79]. The pooled prevalence estimates from in this analysis further illustrate these disparities: 64.8% in low-income and 47.8% in lower-middle-income countries [79], underscoring the need for tailored healthcare policies to address NSPC in resource-limited settings.

The findings of our analysis indicate that younger age, poverty, and cohabitation with other young children are associated with increased NSPC, especially in low-income countries. Addressing these disparities requires focused healthcare policies and vaccination strategies to mitigate NSPC rates and improve pneumococcal disease prevention. Ongoing surveillance and cost-benefit evaluations of broader PCV20 implementation could inform future vaccine strategies and ensure continued protection against both vaccine and non-vaccine serotypes.

Several limitations of this meta-analysis must be noted. First, the variability in study designs, sample sizes, and geographical contexts introduces heterogeneity that may impact the findings' generalizability. Additionally, differences in laboratory methods for NSPC detection across studies could influence reported prevalence rates. Lastly, this review did not analyze serotype distribution within Streptococcus *pneumoniae*, which is crucial for future vaccine strategy development. Understanding the prevalence of specific serotypes is essential to tailoring pneumococcal vaccine formulations effectively, especially for low-income regions where serotype diversity may significantly influence vaccine effectiveness.
